# An integrated treatment delivery system for CSRS and CSRT and clinical applications

**DOI:** 10.1120/jacmp.v4i4.2496

**Published:** 2003-09-01

**Authors:** A. Shiu, B. Parker, J.‐S. Ye, J. Lii

**Affiliations:** ^1^ Department of Radiation Physics The University of Texas M. D. Anderson Cancer Center 1515 Holcombe Boulevard Houston Texas 77030

**Keywords:** conformal stereotactic radiosurgery and radiotherapy, integration

## Abstract

An integrated treatment delivery system for conformal stereotactic radiosurgery (CSRS) and radiotherapy (CSRT) has been developed through a collaboration involving Siemens Medical Systems, Inc., Tyco/Radionics, Inc., and The University of Texas M. D. Anderson Cancer Center. The system consists of a 6‐MV linear accelerator (LINAC) equipped with a Tyco/Radionics miniature multileaf collimator (mMLC). For the conventional SRS treatment, the circular collimator housing can be attached to the opening window of the mMLC. The treatment delivery system is integrated with a radiotherapy treatment planning system and a record‐and‐verify system. The purpose of this study is to report the characteristics, performance, benefits, and the clinical applications of this delivery system. The technical specifications of the LINAC and mMLC were tested, and all the specifications were met. The 80% to 20% penumbral width for each mMLC leaf is approximately 3 mm and is nearly independent of the off‐axis positions of a leaf. The maximum interleaf leakage is 1.4% (1.1% on average) and the maximum intra‐leaf leakage is 1.0% (0.9% on average). The leaf position precision is better than 0.5 mm for all the leaves. The integration of the SRS/SRT treatment planning system, mMLC, and LINAC has been evaluated successfully for transferring the patient treatment data file through radiotherapy treatment planning system to the patient information and treatment record‐and‐verify server and the mMLC controller. Subsequently, the auto‐sequential treatment delivery for SRS, CSRS/CSRT, and the step‐and‐shoot intensity‐modulated radiotherapy has also been tested successfully. The accuracy of dose delivery was evaluated for a 2‐cm spherical target in a Radiological Physics Center SRS head phantom with GAFChromic films and TLD. Five non‐coplanar arcs, using a 2‐cm diameter circular collimator, were used for this simulation treatment. The accuracy to aim the center of the spherical target was within 0.5 mm and the deviation of dose delivery to the isocenter of the target was within 2% of the calculated dose. For the irregularly shaped tumor, a tissue‐equivalent head phantom was used to evaluate the accuracy of dose delivery for using either geometric conformal treatment or IMRT The accuracy of dose delivery to the isocenter was within 2% and 3% of the calculated dose, respectively. From October 26, 1999 to September 30, 2002, we treated over 400 SRS patients and 70 SRT patients. Four representative cases are presented to illustrate the capabilities of this dedicated unit in performing conventional SRS, CSRS, and CSRT. For all the cases, the geometric conformal‐plan dose distributions showed a high degree of conformity to the target shape. The degree of conformity can be evaluated using the target‐volume‐ratio (TVR). Our preferred TVR values for highly conformed dose distributions range from 1.6 to 2.0. The patient setup reproducibility for the Gill‐Thomas‐Cosman (GTC) noninvasive head frame ranges from 0.5 to 1 mm, and the head and neck noninvasive frame is within 2 mm. The integrated treatment delivery system offers excellent conformation for complicated planning target volumes with the stereotactic setup approach, ensuring that dose delivery can be achieved within the specified accuracy. In addition, the treatment time is comparable with that of single isocenter multiple‐arc treatments.

PACS number(s): 87.53.Kn, 87.53.Ly

## I. INTRODUCTION

The current technology of linear accelerator (LINAC)‐based stereotactic radiosurgery (SRS) as practiced in most institutions around the world is based on three‐dimensional cross firing of the target by multiple‐arc beams.^1^ ^,^ ^2^ This methodology delivers a spherical homogeneous treatment volume with a very rapid fall off in dose outside of the target volume. Such treatment is most applicable for treatment of small tumors that resemble spheres. In instances of less ideally shaped tumors, the treatment volume will include a larger proportion of healthy brain. Hence, treatment with a larger circular collimator exposes more normal brain to high‐dose irradiation. For this reason doses prescribed for delivery with larger collimators are lower than those prescribed for delivery with smaller collimators. Therefore, volumes larger than 4 cm in diameter are not even considered for SRS. To better conform to an irregular tumor volume, two or more overlapping isocenters can be used. This leads to hot areas within the overlap region and increased complications.^3^ It is clear that brain toxicity can be reduced by conforming the irradiated volume to the target volume and by maintaining the dose distribution within the target as uniformly as possible.^4^ ^–^ ^10^ This goal can now be achieved with the development of the miniature multileaf collimator (mMLC)^8^ ^,^ ^9^ in The Radiation Physics Department of The University Texas M. D. Anderson Cancer Center. In 1998, we have worked with vendors (Siemens Medical Systems, Inc., Concord, CA, and Tyco/Radionics, Inc., Burlington, MA) to develop a dedicated integrated delivery system for CSRS and CSRT A similar designed micro‐MLC (BrainLab AG, Heimstetten Germany) was commissioned and reported by Xia *et al*.^11^ in 1999.

## II. MATERIALS AND METHODS

### A. Linear accelerator

The PRIMART LINAC was chosen because it was developed by Siemens as their intensity‐modulated radiotherapy (IMRT) machine. Its configuration allows easier integration of the mMLC into this treatment delivery system. In addition, it is compatible with the IMPAC database and information system (IMPAC Medical Systems, Inc., Mountain View, CA). The major specifications for the PRIMART are the mechanical stabilities and the beam characteristics, which were evaluated during the LINAC acceptance test.

The mechanical stability of the LINAC is critical for stereotactic irradiation and can be defined as the stability of the isocenter and its position in the patient as a function of the mechanical and beam parameters. The isocenter is the intersection of three rotational axes: collimator, gantry, and couch. The alignment and stability under dynamic rotation of these three axes determine the precision with which the isocenter's position can be defined in the treatment space. The tolerance of mechanical isocenter precision is within 0.5 mm. The stability of the radiation beam is a function of the alignment of the radiation beam to the mechanical collimator axis and the stability of the radiation beam under dynamic motion. The tolerance of radiation isocenter precision is within 0.5 mm. The mechanical isocenter and the radiation isocenter will not be separated by more than 0.5 mm. The three‐shot film‐test technique is used to verify the accuracy of radiation isocenter for the circular collimator and mMLC.

When the mMLC is attached to the PRIMART collimator, this dedicated SRS/SRT LINAC has an interlock to disable the beam ready if the photon jaw settings are greater than 12.2cm×14.4 cm. The central‐axis percent depth dose at a depth of 10 cm will be 67±1% for a 10−cm×10−cm field at a 100‐cm source‐to‐skin distance. The beam flatness and symmetry for a 10−cm×10−cm field without the mMLC shall be within 2% at a 10‐cm depth for the area 2 cm inside the geometric field edge. All the measurements were made with a IC04 ionization chamber (Scanditronix‐Wellhöfer Dosimetrie, Schwarzenbruck, Germany).

### B. Miniature multileaf collimator

The mMLC is a second generation of M. D. Anderson mMLC The mMLC consists of 62 independent tungsten leaves set in two banks (Bank *A* and Bank *B*) of 31, sandwiched in two tungsten blocks. Each leaf is 7 cm thick equivalent to about seven half‐value layers for 6 MV photons. Projected to the isocenter, a leaf has a width of 4.35 mm instead of 4.0 mm. The reason is that the x‐ray target to the bottom of the mMLC leaf distance is now 62.5 cm instead of 67.5 cm. The increased gap from the bottom of collimator housing to LINAC isocenter allowing us to extend the stereotactic technique from intracranial lesions to extracranial sites (e.g., head and neck, lung, liver, spinal cord, and prostate). Therefore, the maximum field size of mMLC is 13.5cm×10.8 cm at isocenter. The leaves travel straight on a plane with leaf faces are either step down (Bank *A*) or step up (Bank *B*) with step width of 0.25 mm, which allows any pair of leaves to completely close to minimize the radiation leakage through the leaf face end. However, the interleaf surfaces of all leaves are fitted with tongue and groove with each other and the ray‐lines through the interleaf surface are converged to the x‐ray target. The leaves are positioned in the field by small dc motors. Each leaf could be extended to positions whose values ranged from an opening extending 5.4 cm from the field centerline to 5.0 cm crossing the field centerline. In addition, the current circular collimators are available as an option for SRS.

**Figure 1:**
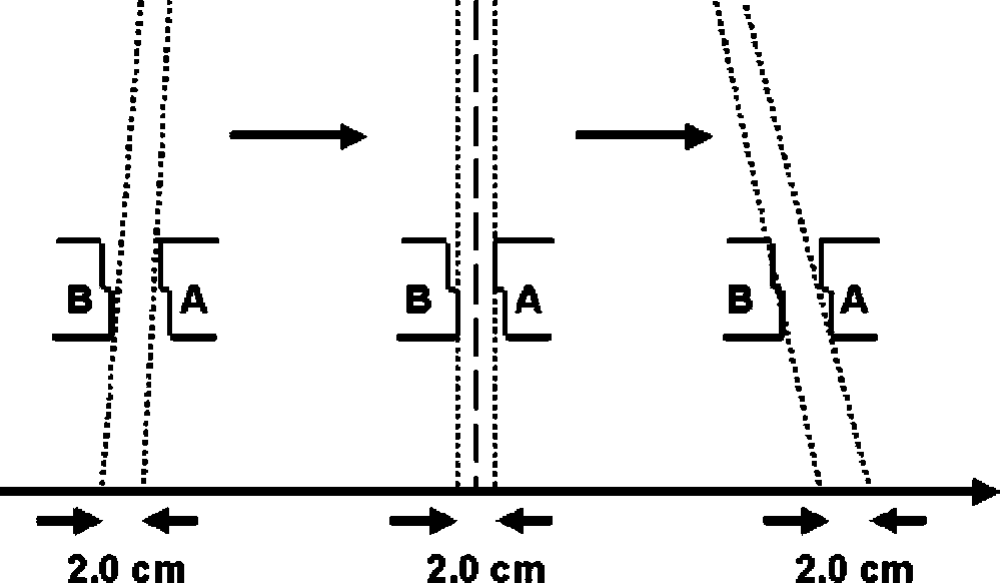
Illustration of a 2.0–cm×12.6–cm field moved asymmetrically over its full range of travel.

An experiment was designed to evaluate the 80% to 20% penumbral width for each mMLC leaf at different off‐axis positions (Fig. 1). A 2.0–cm×12.6–cm field strip is formed with the leaves of bank B at +4 cm away from the central axis and the leaves of bank *A* at −2 cm crossover from the central axis. Subsequently, the 2‐cm strips were at the (2,0), (0,2), and (−2,4) off‐axis positions respectively. The measurements were made in a solid water phantom using therapy localization, and Kodak XTL films (Eastman Kodak, Rochester, NY) at depths of 1.5 and 10.0 cm, respectively. The film was exposed for 4 and 6 monitor units (MU) respectively, because XTL film response is approximately linear from 0 to 5 cGy.^12^ Each of the profiles was normalized to the central axis value of that depth.

The inter‐leaf leakage (between the leaves and the leaf ends) and the intra‐leaf leakage (transmission through the height of the leaves) were also measured using XTL film. A sheet of XTL film was placed at isocenter perpendicular to the beam with 1.5‐cm solid water for buildup. The film rested on a stack of 30‐cm‐thick Styrofoam. The solid water was used to provide enough buildup to reach electronic equilibrium, and the Styrofoam was used to minimize the backscattered radiation from the table and the floor. A 10–cm×12–cm open field was set, and the film was exposed for 4 MU. All of the leaves were fully closed with the leaf‐end junction located at −2 cm with respect to the *A* bank of leaves, and 250 MU were delivered. This was repeated for leaf‐end junctions located at 0 cm and +2 cm with respect to the *A* bank of leaves. All the film readings are normalized to the film reading at the central axis of the 10–cm×12–cm open field and are also corrected for the MU difference by dividing the ratio by 62.5 (=4/250).

### C. TYCO/RADIONICS mMLC/SIEMENS PRIMART integration

Either geometric field shaping or IMRT that produces the prescribed dose conformal to the target volume can be used to reduce dose to normal tissue while minimizing the dose inhomogeneity within the target volume. As we all know, the treatment settings can be very complicated. It is essential to be able to electronically download all the treatment parameters, including a single leaf setting file and dose prescription from the treatment planning system to the patient database and treatment record and verify system. This approach will greatly reduce human error that is possible when entering the wrong treatment parameters, especially for IMRT. In addition, after the patient setup is verified, the treatment delivery system will be capable of automatically delivering a set of treatment fields.

### D. Phantom studies for dose delivery verification

**Figure 2:**
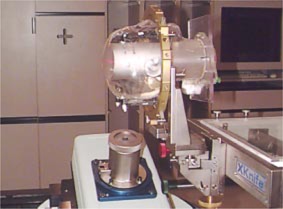
(Color) The circular collimator is attached to the opening window of the mMLC.

The Radiological Physics Center (RPC) head phantom was used to evaluate the accuracy of the whole process of SRS treatment using a 2‐cm diameter circular collimator.^13^ The treatment planning was performed for treating a 2‐cm spherical target inside the RPC head phantom. Five non‐coplanar arcs were used for this simulation treatment to deliver a minimum dose of 20 Gy to the spherical target. The radiochromic (GAFChromic MD 55) films and TLD were used to measure three major axes profiles and dose at isocenter, respectively. An *H‐D* curve for the GAFChromic film up to 30 Gy with 2.5 Gy increments was also measured. The Brown‐Roberts‐Well (BRW) head frame was used for the RPC head phantom and is shown in Fig. 2. The circular collimator housing is attached to the opening window of the mMLC. When the circular collimator was used for this study, the mMLC was completely retracted and at park position. The photon jaws were set at 6 cm×6 cm.

**Figure 3:**
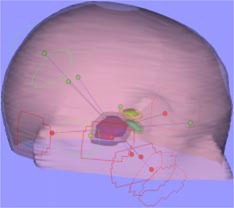
(Color) Display of the entrance and exit shaped fields. CTV (maroon) and the expansion of the CTV (shaded area: PTV), optic nerve (green) and optic chiasm (yellow‐green), and the expanded areas outside the critical structures.

A tissue equivalent head phantom with an irregularly shaped target near the optic nerve and the optic chiasm, was made by Antes to compare the mMLC and the circular collimators for stereotactic SRS or SRT.^8^ We used this head phantom to evaluate the accuracy of dose delivery using non‐coplanar geometric conformal fields or IMRT. Six fields were used for either geometric conformal treatment or IMRT treatment. The six geometric conformal fields are shown in Fig. 3 (green shapes, entrance fields; red shapes, exit fields). The clinical target volume (CTV) in maroon and the planning target volume (PTV) to include the setup uncertainty and the optic nerve in green and the optic chiasm in yellow‐green are also shown in Fig. 3. The dose of 10 Gy was planned for the PTV for both geometric conformal treatment and IMRT treatment. The radiochromic (GAFChromic MD 55) films were also used for the multiple axial planes dose measurement. (Note: The signal is too noisy for GAFChromic film exposed to the dose below 3 Gy. Therefore, we doubled the PTV planned dose (from 10 to 20 Gy) for the simulation treatment of conformal treatment and IMRT.

### E. Clinical applications

Since October 26, 1999, the dedicated machine has been available for clinical use for SRS/SRT To date, we have treated over 400 SRS patients and 70 SRT patients. Four representative cases treated with mMLC will be discussed. The Brown‐Roberts‐Well (BRW) head frame was used as the fixation and target localization device for the SRS case, the Gill‐Thomas‐Cosman (GTC) noninvasive head frame was used for two SRT cases, and the noninvasive head and neck frame was also used for a SRT case. The patient treated with SRS has an elongated brain metastasis with primary renal cell carcinoma. The prescribed total dose to the tumor was 14 Gy in a single fraction. One of the GTC patients has a brain metastasis with unknown primary. The lesion is adjacent to the brain stem and near the optic chiasm. The prescription was 25 Gy in five fractions. The other GTC patient was referred to us for recurrent nasopharynx carcinoma. In a previous treatment, the patient received 70 Gy total dose. In this retreatment, the prescribed total dose to the gross tumor was 70 Gy per 35 fractions. The diagnosis for the noninvasive head and neck patient is non‐Hodgkin lymphoma. The patient had surgery and her nasopharynx was removed. We are treating the cavity with a 0.5‐cm margin to 39.6 Gy for 22 fractions.

## III. RESULTS AND DISCUSSION

### A. Linear accelerator

The PRIMART (LEXAR) and mMLC were accepted on September 27, 1999. All acceptance measurements were within specifications. Some of the results are: (1) the radiation isocenter is within 0.5 mm for the circular applicator and mMLC; (2) the depth of maximum dose $\left(D_{100}\right)$ along the central axis is 1.5 cm, and the depth dose at 10 cm is 67.4% of $D_{100}$; and (3) the beam flatness at 10 cm for the cross‐plane profile is +0.1% to −1.7% and in‐plane is +0.2%/−1.3%. Both are normalized with respect to the dose at central axis for the same depth.

### B. Miniature multileaf collimator

The penumbra value (80% to 20% width) for each mMLC leaf is around 3 mm, which is about 0.3 mm wider than the penumbra from the original design. The penumbra value is almost independent of the off‐axis positions of a leaf. The interleaf leakage (between the leaf and the leaf ends) was found to be 1.4% maximum (1.1% average), whereas the maximum intra‐leaf leakage (transmission through the height of the leaves) was 1.0% (0.9% average). The match‐lines between the abutting fields were found to be straight and of uniform width with less than 0.4 mm variation. The leaf position precision was found to be better than 0.5 mm for all the leaves, which was reported in a previously published paper.^14^

### C. Tyco/Radionics mMLC/Siemens PRIMART integration

**Figure 4:**
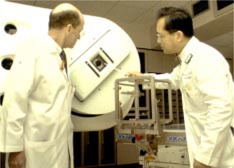
(Color) The mMLC is a part of the PRIMART LINAC.

**Figure 5:**
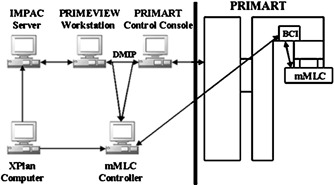
The overall system diagram of the XPlan, mMLC, and PRIMART integration, the mMLC controller identifies the mMLC code and the patient ID from DMIP, which is the Digital Mevatron Interface Protocol using as the setup guidelines for the data to be transferred through serial communication lines. When the mMLC controller has received the mMLC code to set a mMLC field successfully, the mMLC controller sets a value in the block code interface (BCI) box inside the LINAC If the PRIMART control console reads the value and it corresponds to the anticipated value, it allows the treatment to proceed. The BCI box serves as the integration link between the mMLC and the LINAC.

The mMLC is integrated as part of the PRIMART, as shown in Fig. 4. The integration among the XPlan treatment planning system (Tyco/Radionics, Inc., Burlington, MA), mMLC, and the PRIMART has been tested, and the integration was achieved completely now. Figure 5 illustrates the flow of data through this integration process. This process consists of two phases. First is the planning phase, the patient is created in the ACCESS system through the IMPAC server (IMPAC Medical Systems, Inc., Mountain View, CA) and is planned using the XPlan treatment planning system. The treatment data file is transferred via file‐transferred procedure (FTP) to the IMPAC ACCESS. Then, the treatment data file for that patient is imported to PrimeView computer (Siemens Medical Systems, Concord, CA). The treatment data file includes such information as the mMLC code, field shape code, jaw‐setting interlock (jaws not to exceed 12.2× 14.4 cm), monitor units, gantry position, collimator position, couch positions, setup tolerance, etc. The sequence of the mMLC field, the mMLC leaf positioning with unique mMLC block code specifically for that field, and patient name and identification number are exported to the mMLC controller from XPlan.

In the treatment phase, the treatment is downloaded from IMPAC ACCESS via PrimeView into the PRIMART console. As shown in the diagram, serial communication lines (Digital Mevatron Interface Protocol) are monitored by the mMLC computer and alert the mMLC controller software when a mMLC treatment is to begin. Data on these lines specify the patient and field (within the treatment plan). The mMLC controller locates the treatment file and sets the specified field. Subsequent fields for the same patient are set from the same treatment file.

When a field is set successfully, the mMLC controller sets a value in the block code interface box inside the LINAC. The Siemens software reads this value and, if it corresponds to the anticipated value, allows treatment to proceed. At this moment, the console displays that all treatment‐preset parameters match the actual parameters. The user presses “Accept” driving the system to the “Ready State.” There is a different value for each field in the treatment. While the mMLC is in the process of setting a field (leaves are moving, correct position not confirmed), a special value is set; this value indicates that the mMLC is “Not Ready.” Treatment cannot proceed until this value is set. During the treatment, the power to the motor of each leaf is interrupted until the treatment for this field is completed. After treatment completion, the console will upload treatment parameters.

In the case of an IMRT treatment, a group of segments will be created in PrimeView. Each segment will have a specific block code. The console will be designed to allow a pause during beam block changes performed by the mMLC controller and the block code interface. During pause and while the block code is changed and the mMLC leaves are moving, the interlock will remain activated.

Acquisition of beam data for treatment planning, calibration, quality assurance procedures and patient alignment procedures were completed on October 23, 1999.

### D. Phantom studies for dose delivery verification

The results from three major axis profile measurements revealed that the accuracy to aim the center of the 2 cm spherical target was within 0.5 mm, and the deviation of dose delivery to the isocenter of the target was within 2% of the calculated dose.

**Figure 6:**
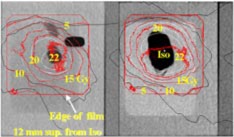
(Color) Comparisons of measured (in red) vs calculated axial dose distributions at plane 12 mm superior from isocenter and at isocenter plane from six non‐coplanar conformal fields.

The results for the six non‐coplanar geometric conformal fields indicated the isocenter dose obtained from GAFChromic film was within 2% of the calculated isocenter dose (22.4 Gy). The comparisons of axial dose distributions between the measurement in red and the calculation in white and black at a plane of 12 mm superior from isocenter and at the plane through isocenter are shown in Fig. 6. The comparisons between the measured and calculated isodose contours agree well except the 22 Gy isodose line.

**Figure 7:**
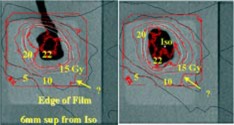
(Color) Comparisons of measured (in red) vs calculated axial dose distributions at plane 12 mm superior from isocenter and at isocenter plane from six non‐coplanar IMRT beams.

The results from the IMRT treatment indicated that the isocenter dose obtained from GAFChromic film was within 3% of the calculated isocenter dose (22.4 Gy). The comparisons of axial dose distributions between the measurement in red and the calculation in black and white at a plane of 6 mm superior from isocenter and at the plane through isocenter are shown in Fig. 7. The comparisons between the measured and calculated isodose contours agree well, except the 22 Gy isodose line. In addition, the measured 10 Gy isodose line at the lower corner was quite deviant from the calculated isodose. We need to make further investigation to resolve the difference.

### E. Clinical applications

#### 1. Case 1: Brain metastasis (SRS)

**Figure 8:**
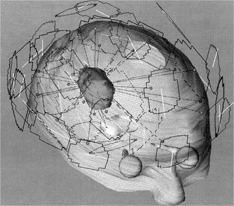
Display of 15 entering shaped fields in four couch positions: the target volume, brain stem, eyes, and optic nerves.

**Figure 9:**
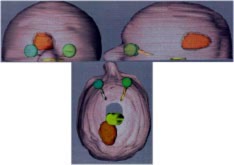
(Color) Display of the 90% iso‐surface dose cloud (orange color) conformed to the projected target volume on anterior, left lateral, and vertical views, respectively.

The first patient case is the recurrent brain metastasis at the right parietooccipital lobe originally from renal cell carcinoma. The tumor volume was 27.2 cm^3^. A treatment plan was generated using 15 geometric conformal fields with four couch positions. The bird's‐eye views of mMLC fields for the SRS treatment focused on the tumor (red) are depicted in Fig. 8. The total dose of 14 Gy was prescribed to 90% of the dose to isocenter. A treatment volume ratio (TVR) of 1.6 was achieved with this plan. The TVR is the total volume receiving the prescribed dose (inclusive of the tumor) divided by the tumor volume receiving the same dose. This statistic can be used as a measure of the conformity of the dose distribution to the tumor volume. It is important to note that if we use a circular collimator for this treatment, it will require at least four isocenters to cover the targeted lesion. However, the actual mMLC conformal field plan utilized only a single isocenter. The 90% iso‐surface dose (orange) is highly conformal to the projected target volume on the anterior, lateral, and vertical views, respectively, as shown in Fig. 9. This treatment not only reduced the lethal dose to the normal brain tissue, but also reduced the treatment time from 3 h (three to four isocenter irradiations) to 30 mins.

#### 2. Case 2: Brain metastasis (SRT)

**Figure 10:**
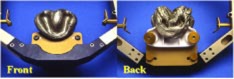
(Color) The front and back view of a customized bite block attached to a GTC frame for a patient is edentulous.

**Figure 11:**
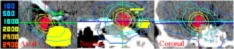
(Color) Display of isodose distributions on axial, sagittal, and coronal planes.

The second example is a brain metastasis with unknown primary. The treatment plan was generated using seven noncoplanar‐shaped fields to deliver 25 Gy in five fractions to the lesion. The patient was immobilized with a GTC frame. Because the patient is edentulous, a customized bite block was made by the dentistry within the head and neck surgery department (Fig. 10). The patient had been able to wear the frame reproducibly, and the stereotactic treatment reproducibility was within 1 mm, which agreed well with previous reports.^14^ ^–^ ^16^ The isodose distributions are shown in the axial‐, sagittal‐, and coronal‐planes, respectively, in Fig. 11. The prescribed dose (25 Gy), which was normalized to the 90% of the dose at isocenter, conformed well to the projected target area. The TVR of 2.0 was achieved with this plan. The maximum dose to the brainstem is 25 Gy, but only 2% of the brainstem received a dose greater than 20 Gy. The dose to the optic nerves and chiasm were all far below their tolerance doses.

#### 3. Case 3: Recurrent nasopharynx carcinoma (SRT)

**Figure 12:**
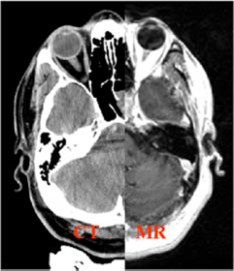
A planning CT/MR fusion axial image shows the closeness of the lesion to the left optic nerve and the optic chiasm.

**Figure 13:**
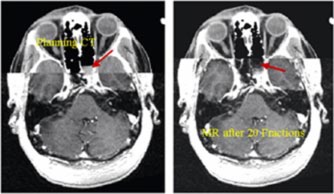
A sample of MR image obtained after 20 out of 35 treatments is fused with the planning CT image. The tumor is seen on the planning CT image, but is almost invisible on the MR image as shown by the red arrow.

The third case is that of a recurrent mass filling the sphenoid sinus mostly on the left with possible invasion into left cavernous sinus. Because the lesion was adjacent to the left optic nerve and also very close to the optic chiasm, as shown in Fig. 12, the patient had a significant risk of left optic neuropathy. The patient also had a low probability but real risk of neuropathy for the optic chiasm. Previous treatment to the nasopharynx with a prescription dose of 70 Gy in 35 fractions creates a great challenge to produce an optimal plan to deliver an additional 70 Gy in 35 fractions. A six‐beam noncoplanar‐shaped field plan was generated for this treatment. Although the optic nerves and chiasm were outside the previous treatment volume, the total dose of 70 Gy was prescribed to 95% of the dose at isocenter. The TVR of 2.3 was achieved for this plan although it was outside our preference range; however, the H&N Radiation Oncologist accepts it. The maximum dose to the optic chiasm was below 47 Gy, and the maximum dose to the right optic nerve was below 45 Gy. The maximum dose to the left optic nerve was 60.8 Gy. Less than 2% left optic nerve volume received 60 Gy. Therefore, 10% of the tumor adjacent to the left optic nerve received the dose between 62 to 70 Gy. The reproducibility of the GTC frame for this setup was within 0.5 mm, which was verified from two anterior and Lat portal films per week. A MR image set was obtained after 20 out of 35 treatments and a sample slice of the fused image is shown in Fig. 13. The enhancement of the lesion was observed on the planning CT image, but not on the MR image. The patient remains recurrence free up to date (four years post treatment) and has reported no vision problems.

#### 4. Case 4: A diffuse large cell lymphoma (SRT)

**Figure 14:**
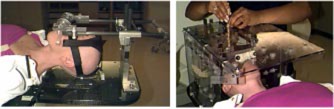
(Color) Display of a Tyco/Radionics noninvasive H&N frame (left) and an H&N localizer box, which is attached to an H&N baseboard (right). This localizer not only provides the spatial coordinates for the target with respect to the frame but also serves to reposition the patient in a specific location for multiple fractionated treatment.

The fourth case is that of a diffuse large cell lymphoma involving the nasopharynx. The treatment plan includes six noncoplanar fields shaped to the nasopharyngeal access. The plan used the 6 MV photons and delivered a total dose of 39.6 Gy in 22 fractions. The patient was immobilized with the Tyco/Radionics noninvasive head and neck (H&N) stereotactic frame (Fig. 14). This frame immobilized the patient's head and neck for SRT. Prior to obtaining a CT image set for planning, a H&N localizer box was attached to a H&N baseboard. The depth probes are inserted through the guide tubes to realign the patient. The mean difference between the daily setup versus the initial setup should be within 1 mm. In addition, three tattoos (one at forehead and two on the lateral areas of the head) are used to minimize the head rotation. During the CT scan, all seven rods were seen on all axial slices, providing the information needed to localize any point spatially within the H&N frame space defined by the localizer box. The reproducibility of the H&N frame for this setup was within 2 mm, which was verified from the orthogonal portal films taken twice per week.

**Figure 15:**
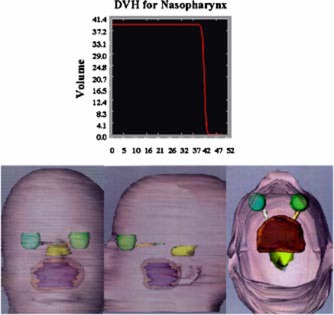
(Color) Display of the integral dose volume histogram and the 39.6 Gy iso‐surface dose (orange), which is highly conformed to the projected target volume on the anterior, lateral, and vertical views, respectively.

Figure 15 shows the integral dose volume histogram and the 39.6 Gy iso‐surface dose (orange), which is highly conformed to the projected target volume on the anterior, lateral, and vertical views, respectively. A TVR of 2.0 was achieved for this plan. The minimum dose to the target was 39.6 Gy, which is normalized to 95% of the dose at isocenter and the maximum dose was 43 Gy.

## IV. CONCLUSIONS

An integrated treatment delivery system has been developed through collaboration of Siemens, Tyco/Radionics, and M. D. Anderson Cancer Center. All the technical specifications of the LINAC and mMLC have met the necessary requirements. The integration among the SRS/SRT treatment planning system, mMLC, and LINAC has been tested successfully for transferring the patient treatment data file through radiotherapy treatment planning system to the patient information and treatment record and verify server and the mMLC controller. The treatment delivery system is capable of using auto‐sequential treatment delivery for SRS, CSRS/CSRT, and step‐and‐shoot IMRT.

The simulated SRS treatment using a circular collimator to a RPC head phantom demonstrates that the accuracy of the alignment and the dose delivery for a whole SRS process is very well within the requirements for this procedure (alignment, 1 mm; isocenter dose, 3%). The simulated CSRS and IMRT treatments demonstrated the doses delivered to an irregularly shaped target were accurate and the measured axial dose distributions compared well with the calculated dose distributions.

Four examples are presented here to demonstrate the potential benefits of using mMLC for CSRS and CSRT treatments of brain lesions and head and neck lesions. For all the cases, the geometric conformal plans showed a high degree of conformity of the dose distribution with the target shape, which can be evaluated using the TVR. Our preferred TVR values for highly conformed dose distributions range from 1.6 to 2.0. The patient setup reproducibility for the GTC noninvasive head frame ranges from 0.5 to 1 mm, within 2 mm for the head and neck noninvasive frame. This paper demonstrates that the integrated treatment delivery system offers excellent conformation for complicated planning target volumes, with the stereotactic setup approach ensuring dose delivery within the specified accuracy. In addition, the treatment time is comparable with that of single‐isocenter, multiple‐arc treatment. This paper also demonstrates that an integrated treatment delivery system can safely and effectively provide auto‐sequential treatment delivery for SRS, CSRS/CSRT, and step‐and‐shoot IMRT.
